# Data Integration for Microarrays: Enhanced Inference for Gene Regulatory Networks

**DOI:** 10.3390/microarrays4020255

**Published:** 2015-05-14

**Authors:** Alina Sîrbu, Martin Crane, Heather J. Ruskin

**Affiliations:** 1 Department of Computer Science and Engineering, University of Bologna, Via Mura Anteo Zamboni 7, Bologna 40126, Italy; 2 Center for Scientific Computing and Complex Systems Modelling, School of Computing, Dublin City University, Glasnevin, Dublin 9, Ireland; E-Mails: mcrane@computing.dcu.ie (M.C.); hruskin@computing.dcu.ie (H.J.R.)

**Keywords:** data integration, microarrays, gene regulatory networks, transcriptional regulation, reverse engineering

## Abstract

Microarray technologies have been the basis of numerous important findings regarding gene expression in the few last decades. Studies have generated large amounts of data describing various processes, which, due to the existence of public databases, are widely available for further analysis. Given their lower cost and higher maturity compared to newer sequencing technologies, these data continue to be produced, even though data quality has been the subject of some debate. However, given the large volume of data generated, integration can help overcome some issues related, e.g., to noise or reduced time resolution, while providing additional insight on features not directly addressed by sequencing methods. Here, we present an integration test case based on public *Drosophila melanogaster* datasets (gene expression, binding site affinities, known interactions). Using an evolutionary computation framework, we show how integration can enhance the ability to recover transcriptional gene regulatory networks from these data, as well as indicating which data types are more important for quantitative and qualitative network inference. Our results show a clear improvement in performance when multiple datasets are integrated, indicating that microarray data will remain a valuable and viable resource for some time to come.

## 1. Introduction

Gene regulatory networks (GRNs) are an important mechanism through which cells control gene expression. Cellular network discovery [1] and, in particular, GRN discovery are major aims of systems biology, as they provide valuable knowledge on biological processes, which may help to uncover disease markers and to design novel treatments [2]. GRNs may include interactions between transcription factors and genes (transcriptional regulation), but also protein-protein interactions (PPI) (combinatorial regulation from multiple transcription factors or co-regulated genes) [3,4]. Here, however, we concentrate on direct transcriptional interactions only, so modelling transcriptional GRNs.

Various GRN models have been developed over recent years (e.g., [2,5,6,7,8]), facilitated by the advent of gene expression measurement technologies, such as microarrays [9] and, more recently, RNA-seq [10]. These methods measure mRNA concentrations of thousands of genes at the same time, which can be used to reverse engineer the network of transcriptional interactions. Qualitative approaches concentrate on discovering whether pairs of genes interact (e.g., rule-based methods [11], Boolean networks [12] and mutual information [5,13,14], where a discussion on the use of mutual information in microarray data analysis in general can be found in [15,16]). Quantitative techniques aim at building models of regulation, namely systems of differential equations (e.g., [17,18]) or artificial neural networks (e.g., [19]). Qualitative approaches have the advantage of providing a suitable overview for large GRNs, while quantitative methods provide more detailed information on interactions between genes and allow for continuous *in silico* simulation of the expression process, which is important for the analysis of conditions that are difficult to obtain *in vivo* or *in vitro* (e.g., response to treatment).

Quantitative reverse engineering is typically performed using gene expression time series, by minimising the error between real and simulated data. For qualitative methods, any type of data can be used, with a large volume of existing work in this area [5,13]. The main type of gene expression data used remain the same for both types of techniques (*i.e*., microarrays), even if RNA-seq technologies are generating increasingly more data. In this paper, we concentrate on quantitative methods.

At present, quantitative analysis is possible only for a limited number of genes (good performance up to 10 genes [20]), and models do not usually provide a reliable platform for the investigation of real-life scenarios. This is due to factors, such as data noise (discussed in more detail below) and the limited length of the time series, as well as the large number of parameters to be estimated for these models, which typically creates an under-determination problem (with multiple model fits possible). Thus, new criteria for model evaluation and development are needed in order to eliminate some of the possibilities. For the inferential process, this can be achieved by integrating different types of biological data and incorporating additional knowledge, available from public databases.

Currently, data integration is one of the main concerns of systems biology [21] and is expected in the next few years to take reverse engineering of biological networks to a new and higher level [21,22]. In fact, some efforts in this direction have started to appear, although mostly for qualitative models [3,23,24] or with a focus on using one biological dataset combined with previous knowledge [25]. Microarrays are suitable also for quantitative integration, since they continue to provide a cost-effective platform for generating diverse quantitative data, measuring different processes and with online databases available. It is worth remembering that microarray data quality itself has been subject to debate. Sources of noise (microarray design, dyes, probe specificity, cross-hybridisation, image processing) have been shown to affect these data [9], with various normalisation and background subtraction algorithms having been developed to address this. Furthermore, microarray experiments are not always reproducible, as the same sample can produce data variation, providing one reason for the use of technical replicates. Microarrays have also been shown to yield low precision in terms of the exact quantification of gene expression [26]. In particular, very low transcription is affected due to background noise interference [10]. Just as much of a restriction is very high transcription, due to hybridisation saturation (*i.e*., where only a limited amount of cDNA can hybridise to a microarray spot [27]). As such, microarray data analysis is intrinsically likely to benefit from the integration paradigm, since this has been shown to provide a means to decrease noise overfitting [28].

Data integration, however, is not straightforward. Integrating time series from different platforms requires careful pre-processing [29], while inclusion of other types of data needs efficient handling, since increasing the amount of information to be processed can significantly slow down inferential algorithms. In this paper, therefore, we use an integrative tool based on evolutionary computation, EGIA [30] (evolutionary computation for GRNs, an integrative algorithm), in order to analyse several microarray datasets together with other data types. This platform has been shown to compare well with existing GRN inference methods [30]. Developmental time series data for *Drosophila melanogaster* from two sources, together with microarray knock-out experiments and other meta-data on interactions, are combined using two different integration mechanisms—exploration of the possible space of interactions and specific model evaluation. The former enhances the mechanism through which qualitative information on interactions is found (*i.e*., whether two genes interact or not). The latter relates to the evaluation of candidate models, which is an important step in evolutionary optimisation. The importance of each dataset in each mechanism is determined, and a precise strategy for the integration of these data is provided. We show that, when properly performed, integration improves both interaction networks and quantitative simulation capability for our inferential models, when compared to the use of time series alone.

## 2. Materials and Methods

### 2.1. Data

*Drosophila melanogaster* (fruit fly) data have been employed in our integrative analysis, with several types of data retrieved from publicly available databases. These include time series data from two platforms (retrieved from the Gene Expression Omnibus (GEO) database [31]), a set of knock-out (KO) microarray experiments, position-specific weight matrices (PSWMs; [32]), known *cis*-regulatory modules and Gene Ontology (GO) annotations. For model validation, a set of previously known transcriptional interactions extracted from the DROID database (Drosophila Interactions Database; [33]) has been used. A subnetwork of 27 genes involved in embryo development, listed in Table 1, has been analysed. These have been chosen by starting from the main genes involved in segmentation [17], followed by the addition of several genes known to interact with this main set (based on the FlyBase interactions browser [34]).

**Table 1:** Set of 27 genes selected for network analysis for the *Drosophila melanogaster* dataset.

Gene names								
arm	bcd	cad	CrebA	Egfr	en	eve	ftz	fz
gt	hb	hkb	how	ken	Kr	L	Mef2	mxc
noc	os	pnr	ras	smo	sna	Tl	tor	twi

**Dual-channel (DC) dataset.** This time-course dataset analyses gene expression during Fly embryo development, using dual-channel microarrays (GEO Accession GSE14086 [35]). The dataset contains seven time points sampled at 1- and 2-h intervals, up to 10 h after egg laying. Three biological replicates are available, resulting in three time series in total.

**Single-channel (SC) dataset.** The single-channel dataset [36], measured with Affymetrix arrays, contains gene expression measurements for 12 time points during *Drosophila melanogaster* embryo development. Samples have been taken every hour up to 12 and a half hours after egg laying. Three biological replicates are included. Both the SC and DC datasets were normalised using cross-platform normalisation [37], which was shown previously to be a valid option for time series data integration [29].

**Previously known transcriptional interactions (DROID dataset).** For validation purposes, a set of known transcriptional interactions has been retrieved from DROID (Drosophila Interactions Database; [33]), Version 2010_10. This consists of 16 pair-wise interactions between transcription factors and their target genes, for the 27-gene network under analysis. This gold standard was used due to the fact that these interactions are confirmed experimentally. Although it is not the complete network for the 27 genes and it does not include PPIs, it does help to indicate the quality of our models in terms of underlining transcriptional regulation, which is the interest of this study. The exact interactions are included as Supplementary Material.

**KO dataset.** Five KO microarray datasets have been retrieved form the GEO database. These contain knock-out experiments for 8 genes and the corresponding wild-type measurements. The accession numbers for the datasets are GSE23346 ([38], Affymetrix Drosophila Genome 2.0 Array, 6 samples), GSE9889 ([39], Affymetrix Drosophila Genome Array, 20 samples), GSE7772 ([40], Affymetrix Drosophila Genome Array, 4 samples), GSE3854 ([41], Affymetrix Drosophila Genome Array, 54 samples) and GSE14086 ([35], dual-channel array, 63 samples). For these, the log-ratios between knock-out and wild-type expression values have been used within our framework.

**Binding site affinities (BSAs).** A set of PSWMs for 11 transcription factors have been retrieved from [42]. These matrices have been computed using DNA foot-printing data from [43]. In order to compute BSAs using PSWMs, the promoter sequence for each gene is required. For the Drosophila genome, the RedFly database [44] provides a set of known *cis*-regulatory modules, which have been used here for this purpose. *Cis*-regulatory modules for 16 genes have been retrieved, while for the other genes, the upstream 2-Kbp sequence has been used to assess BSA. Using both information types, BSAs were computed for use in our algorithm.

**GO annotations.** GO [45] is a database of genes, which have been annotated to have a specific function or to be involved in specific processes. These annotations come from various sources and have been determined using technologies ranging from those in wet-lab experiments to computational methods. The database is a valuable source of meta-information that can be used in different ways. Here, we have used the GO platform to identify which of the gene products involved in the network analysed have been previously shown to display transcription factor activity.

**Correlations (CORR).** All gene expression data related to the genes of interest were combined, and the Pearson product moment correlation coefficient was computed between gene pairs. These data were fed into the EGIA framework.

### 2.2. GRN Inference

Reverse engineering is performed using the platform EGIA (evolutionary computation for GRNs, an integrative algorithm) [30]. This uses evolutionary optimisation to infer artificial neural network (ANN) models of regulation. The model describes the gene expression level of each gene *i* at a certain time *t* as the output of a sigmoid unit, with input given by the expression values of the gene’s regulators at time t−1:

(1) $$g_{i}\left(t\right)=S\left(\sum_{j\in R_{i}}w_{ij}g_{j}\left(t-1\right)\right)$$

where *S* is the logistic function and $R_{i}$ is the set of regulators of gene *i*, while $w_{ij}$ are the strengths of the effect of gene *j* on gene *i*. The inference problem is divided into two parts: finding the set of regulators $R_{i}$ for each gene and finding the strength of the regulation (parameters $w_{ij}$). The first is solved using a genetic algorithm, which evolves the topology of the GRN. Each topology is evaluated by training the corresponding ANN using time series gene expression data. This training procedure also solves the second problem of finding interaction weights. The training error (assessed by root mean squared error between the real and simulated expression levels) reflects the fitness of the original topology.

The only mandatory type of data for the EGIA platform is time series gene expression data, which give the base fitness of the models. Several measuring platforms and time series can be used, provided these are properly scaled. However, data other than gene expression levels can also be integrated into the optimisation process. The algorithm employs two mechanisms to integrate other data. These accept all types described above or any subset (except for DROID interactions, which are used for model evaluation only).

**Network structure exploration (NSEx).** The EGIA algorithm starts with a set of topologies and permits these to evolve by changing links between genes until better solutions are found. The topologies are obtained by selecting, for each gene, a set of regulators. In a basic genetic algorithm, the selection of the regulators for each gene is random, both when initialising the topologies and when evolving them (initialisation and mutation). EGIA, however, uses non-uniform probability distributions to select regulators, which are based on the additional data. For instance, if a KO experiment shows large log-ratios for a specific gene, this indicates a higher probability link to the silenced gene and increases the probability that this gene is selected as a regulator. Similar mechanisms apply for all data types included, with further details given in [30].

**Network structure evaluation (NSEv).** It is important to include additional data in the exploration of the space of possible structures, as it speeds up the search for models with more realistic interactions. However, it is the final evaluation of the topologies, during the evolutionary process, that decides which solutions are taken to the next generation. A basic algorithm for this would use only the ability of the model to reproduce time series data. However, it may be the case that models with well-established interactions are not complete enough to simulate the data well, so these would be discarded. In order to reduce this effect, when determining the fitness of the topologies, EGIA uses an additional term, which measures how likely a given topology is, based on the NSEx process probabilities, described previously. In this way, all data types available can be integrated. More detail on the implementation can be found in [30].

Both mechanisms above attempt to reduce the under-determination problem for large GRNs. NSEx drives the algorithm more quickly towards useful areas of the search space, while NSEv ensures the longevity of `partially good’ topologies. Hence, the first mechanism can be seen as a guideline only (a weaker integration criterion), while the second is a stronger integration criterion, since it determines the best model. This means that, in order to augment performance, the first mechanism accepts a wider range of data compared to the second.

### 2.3. Analysis

Given the different integration mechanisms available in EGIA, we performed an analysis of possibilities for data integration. The aim here was to evaluate at which stage each dataset is most useful, in order to provide an optimised strategy for integration. Models obtained were evaluated both qualitatively (the topological structure of the GRN, *i.e*., pairwise transcriptional interactions) and quantitatively (the ability to reproduce the evolution of gene expression levels over time).

Qualitatively, the AUROC (area under the ROC curve) and AUPR (area under the precision-recall curve) [46] are computed, using the set of known transcriptional interactions from the DROID data as the gold standard. Given that our algorithm is stochastic in nature and the model quantitative, predictions of interactions have been performed by using multiple models (obtained in different runs) and employing a `voting procedure’ for possible interactions. In this way, an interaction that appears in more models is considered to be more plausible (this method of voting has been previously used to extract qualitative information in similar problems [47,48]). The set of possible interactions is ranked from the highest to lowest number of votes and used for AUROC/AUPR computation. To evaluate the variability of results for the models, we also computed AUROC/AUPR values for subsets of 9 models at a time and reported the standard deviation over all values. This was to enable significant comparison among data integration strategies. Quantitative evaluation of our results refers to the ability of the models to reproduce continuous levels of gene expression over time, through time series simulation. The model starts from the values of gene expression for the first time point in a time series, then evolves independently to generate a simulated series. This is compared with the original data, by computing the RMSE, which gives a quantitative evaluation of the model. In this paper, we employed a cross-validation approach, where the SC time series dataset was used for training and the DC time series for testing. Thus, we started by extracting models from the SC time series dataset, evaluating their ability to simulate quantitative gene expression levels from the DC dataset.

The analysis presented here follows three stages. First, NSEx is employed alone using the available datasets to evaluate whether these are useful for this weaker integration mechanism (Section 3.1). Secondly, NSEv is added to analyse all datasets and to identify the integration scheme with the best results (Section 3.2). Using this scheme, the DC dataset, previously used for quantitative evaluation only, was included in the model extraction phase. The final model thus obtained was qualitatively evaluated only (Section 3.3), since all time series data were used as training data. Table 2 summarises, for the three different analysis stages, how each dataset was used.

**Table 2:** Usage of each dataset at the different analysis stages.

Section	Analysis stage	Mechanism	SC	DC	BSA	KO	Corr	GO	DROID
Section 3.1	Model extraction	Time series	✓						
		NSEx			✓	✓	✓	✓	
		NSEv							
	Model evaluation	Qualitative							✓
		Quantitative		✓					
Section 3.2	Model extraction	Time series	✓						
		NSEx			✓	✓	✓	✓	
		NSEv			✓	✓	✓	✓	
	Model evaluation	Qualitative							✓
		Quantitative		✓					
Section 3.3	Model extraction	Time series	✓	✓					
		NSEx			✓	✓	✓	✓	
		NSEv			✓				
	Model evaluation	Qualitative							✓
		Quantitative							

## 3. Results and Discussion

### 3.1. Integration for NSEx

The first data integration analysed the effects of the NSEx mechanism, which integrates the additional datasets for exploring the structure of the interaction network. In order to identify which dataset is more useful (of those described; Section 2.1), different variants of NSEx were employed to assess the contribution of each data type separately, followed by the integration of all types. Hence, five different variants of NSEx were derived and compared to the algorithms using the SC time series data only: SC+NSEx.KO (using knock-out experiments for NSEx), SC+NSEx.GO (using GO annotations for NSEx), SC+NSEx.BSA (using binding site affinities for NSEx), SC+NSEx.CORR (using correlation among genes for NSEx) and SC+NSEx.ALL (using all data for NSEx).

Table 3 displays AUROC and AUPR values for the five NSEx variants compared to the SC algorithm (using time-series data only). Standard deviations for all values are also included, computed from nine out of ten runs at a time (bootstrapping) and showing very low variability of results. In terms of individual datasets, the set of predicted transcriptional interactions improves when including BSA and KO data, while GO and CORR data seem to have no effect or impact negatively on the interaction quality. However, the combined effect of all datasets does appear to achieve significant improvement in network topology, indicating that even weak data integration has some value, and that collectively, the dataset types can offer enhanced insight.

**Table 3:** Algorithm incorporating NSEx. Qualitative results: AUROC and AUPR values obtained after 10 runs with each algorithm and, in parentheses, standard deviations for subsets of 9 runs (see Section 2.3 for details on how these were computed). Variants: SC (SC time series only, without integration of additional data), SC+NSEx.KO (using knock-out experiments for NSEx), SC+NSEx.GO (using GO annotations for NSEx), SC+NSEx.BSA (using binding site affinities for NSEx), SC+NSEx.CORR (using gene-correlations for NSEx) and SC+NSEx.ALL (using all data for NSEx). For additional datasets, BSA followed by KO lead to improved sets of interactions, while CORR affects selection adversely. However, the combined effect of all data types provides optimal inference of the interaction set.

Algorithm	SC	SC+NSEx.KO	SC+NSEx.GO	SC+NSEx.BSA	SC+NSEx.CORR	SC+NSEx.ALL
AUROC	0.603 (0.017)	0.610 (0.010)	0.593 (0.022)	0.677 (0.021)	0.544 (0.016)	**0.744 (0.018)**
AUPR	0.037 (0.002)	0.045 (0.003)	0.034 (0.002)	0.046 (0.003)	0.036 (0.001)	**0.066 (0.004)**

**Figure 1:**
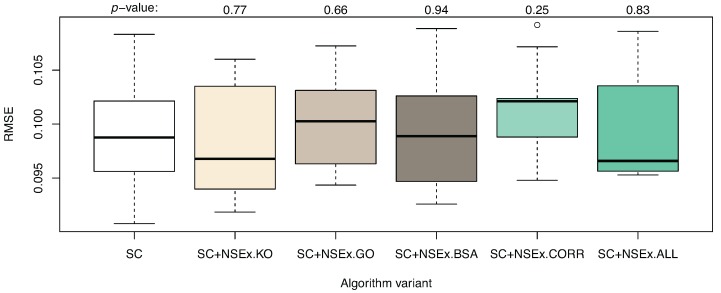
Algorithm enhanced with NSEx: quantitative results. The graph shows the distribution (over 10 runs) of RMSE on test data (DC dataset) for models obtained with algorithm variants (as for Table 3). A *t*-test performed for each enhanced version to compare performance to that of the basic SC variant gave *p*-values as shown. No significant change was observed in RMSE values after integration.

Quantitative analysis was also performed, with RMSE values shown in Figure 1. No improvement in terms of the simulation capability of the models was obtained, although if descriptions of interactions can be improved, so too should gene expression pattern simulation. The current limitations may be due to persistent distortion of the fitness landscape by noise or may be inherently linked to under-determination. The evaluation criterion based on SC data only is crude, so that reverse engineering becomes increasingly fuzzy. This argues strongly for inclusion of further data types in model selection, through the NSEv mechanism.

### 3.2. Integration for NSEv

**Figure 2:**
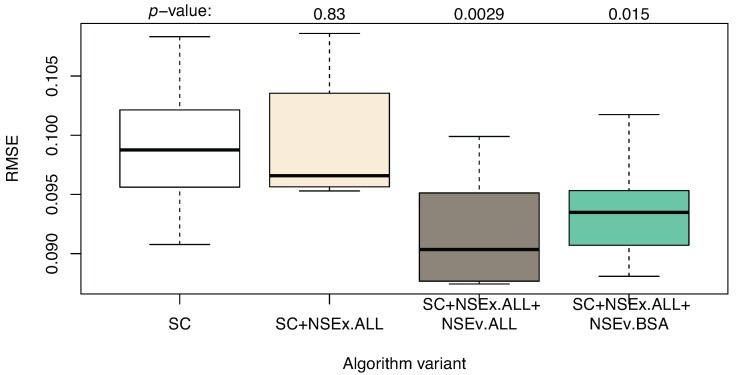
Algorithm enhanced with NSEx and NSEv: quantitative results compared to SC+NSEx and SC only. The variants are: SC (time series only, without integration of additional data), SC+NSEx.ALL (using all data for NSEx), SC+NSEx.ALL+NSEv.ALL (using all data for both NSEx and NSEv) and SC+NSEx.ALL+NSEv.BSA (using all data for NSEx, but BSA only for NSEv). RMSE values show improvement compared to the previous integration strategy; small differences between NSEv.ALL and NSEv.BSA are observed. This suggests that including all data in NSEx scoping with BSA data for refinement in NSEv is optimal.

Having observed an increase in the qualitative value of models for NSEx using all data, we then included the NSEv mechanism (SC+NSEx.ALL+NSEv.ALL) in the algorithm, again using all data available to evaluate model performance (*i.e*., not based on reproduction of SC expression patterns alone). The aim was to decrease quantitative simulation error and provide further improvement in terms of the identification of transcriptional interactions. Figure 2 shows the simulation performance for the models obtained, compared to the SC and SC+NSEx versions of the algorithm. This improves markedly when all data are included in the evaluation and provides some support for our hypothesis that extending the fitness landscape with topological data can improve simulation performance.

Table 4 shows the quality of interactions obtained after integration. In contrast to the error improvement, this appears to decrease compared to the SC+NSEx case, which is surprising considering that the quantitative behaviour improves. This could be due to the fact that the models contain indirect interactions (which might include the PPIs mentioned earlier), enabling good simulation of gene expression levels. However, we are interested in uncovering direct transcriptional interactions. The presence of indirect interactions may be more prominent for certain data types, such as correlation patterns (CORR), KO experiments and GO annotations. While these were filtered out in the case of NSEx (a weak integration criterion), they were forcibly included for the more stringent integration criterion of the NSEv mechanism. Hence, more accurate interactions and maintenance of good simulation performance might be obtained through a hybrid approach using all data for NSEx (*i.e.*, the landscaping step) and only BSA data for NSEv (SC+NSEx.ALL+NSEv.BSA). This refinement is suggested by the fact that BSA data usually indicate direct interactions (the ability of the protein transcription factor to physically bind to the target gene). We tested this hypothesis and indeed found that the best compromise for qualitative and quantitative performance is obtained by using this integration approach, as Table 4 and Figure 2 show.

**Table 4:** Algorithm enhanced with NSEx and NSEv: qualitative results compared to SC+NSEx and SC only. AUROC and AUPR values obtained after 10 runs with each algorithm are shown, together with standard deviations for subsets of 9 runs in parentheses (see Section 2.3). Variants are SC (time series only, without integration of additional data), SC+NSEx.ALL (using all data for NSEx), SC+NSEx.ALL+NSEv.ALL (using all data for both NSEx and NSEv) and SC+NSEx.ALL+NSEv.BSA (using all data for NSEx, but BSA only for NSEv). Integrating all data at the evaluation stage decreases the quality of interactions compared to those obtained with NSEx. Use of BSA alone for evaluation yields better results.

Algorithm	SC	SC+NSEx.ALL	SC+NSEx.ALL+NSEv.ALL	SC+NSEx.ALL+NSEv.BSA
AUROC	0.603 (0.017)	0.744 (0.018)	0.700 (0.027)	**0.764 (0.028)**
AUPR	0.037 (0.002)	0.066 (0.006)	0.049 (0.003)	**0.086 (0.003)**

We also investigated the possibility of eliminating the CORR data from the NSEx mechanism and combining with NSEv.ALL or NSEv.BSA. This is because Table 3 suggests that CORR data are least important for the identification of correct transcriptional interactions. However, the resulting AUROC and AUPR values were not better than the results included in Table 4 for the variants employing CORR data. Specifically, the new AUROC/AUPR values were 0.663/0.054 for NSEv.ALL and 0.763/0.085 for NSEv.BSA. We can thus conclude that although CORR data by themselves do not appear to bring improvement, they are still useful to complement the other datasets. This behaviour was also observed for synthetic data in previous work [30].

### 3.3. Including All Time Series

The analysis presented in the previous section provides a strategy for data integration that optimises both qualitative and quantitative model behaviour. For this, the DC (dual channel) time series data were used only at the `model testing’ stage, to enable quantitative evaluation. However, once the integration strategy is chosen, models can be further refined by integrating both SC and DC datasets in the reverse engineering procedure, which can also reduce noise overfitting [28].

**Figure 3:**
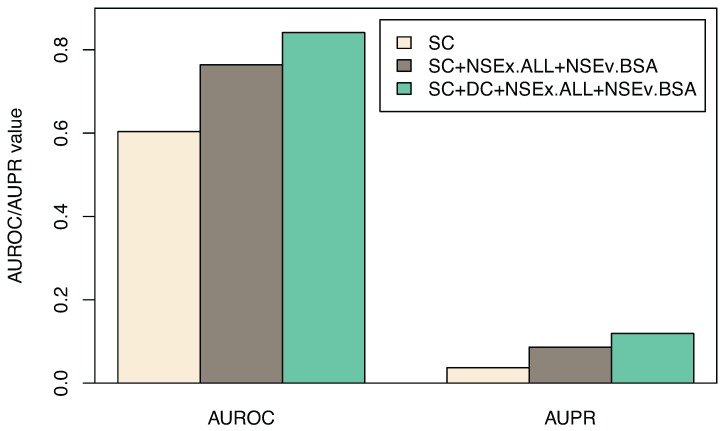
Combining the two time course datasets, SC and DC. AUROC and AUPR values (and standard deviations for subsets of models) for gene connections obtained through integration scheme NSEx.ALL+NSEv.BSA are displayed (see Section 2.3). Shown are the SC dataset alone, SC integration (SC+NSEx.ALL+NSEv.BSA) and SC+DC integration (SC+DC+NSEx.ALL+NSEv.BSA). Overall improvement is ∼20% with the combined data and integration scheme specified.

Finally, therefore, we used the best-performing algorithm variant (NSEx.ALL+NSEv.BSA) to integrate the two microarray datasets (SC and DC) available for the *Drosophila melanogaster* embryo development. The aim was to obtain better prediction of transcriptional interactions between genes. Figure 3 graphically displays AUROC and AUPR obtained by time-series integration, compared to those found using only the SC dataset for training, with exact values shown in Table 5. The increase in AUROC and AUPR values suggests improved prediction of *Drosophila melanogaster* gene interactions predicted from combining the two datasets rather than using one only. Equally, the application of the exact same integration strategy (as before) maintained model simulation performance.

**Table 5:** Combining the two time course datasets, SC and DC. AUROC and AUPR values (and standard deviations) for gene connections obtained through integration scheme NSEx.ALL+NSEv.BSA are displayed. Shown are the SC dataset alone, SC integration (SC+NSEx.ALL+NSEv.BSA) and SC+DC integration (SC+DC+NSEx.ALL+NSEv.BSA).

Algorithm	SC	SC+NSEx.ALL+NSEv.BSA	SC+DC+NSEx.ALL+NSEv.BSA
AUROC	0.603 (0.017)	0.764 (0.023)	**0.841 (0.014)**
AUPR	0.037(0.002)	0.086 (0.003)	**0.119 (0.006)**

## 4. Conclusions

An analysis of data integration for quantitative transcriptional GRN modelling was presented, with a view toward investigating strategies to enhance network model quality and interpretation. A 27-gene network of transcriptional interactions was analysed. We used, as the gold standard, 16 interactions extracted from the DROID database, together with a time series gene expression dataset for the evaluation of simulation abilities. Integration was performed in two stages: exploring the interaction space (NSEx) and evaluating model behaviour (NSEv), through an evolutionary algorithm. The final objective was to provide a robust integration strategy in order to determine the best data type contributions to model performance at each stage.

The qualitative transcriptional gene interaction information was affected most by the integration of different data types in the NSEx stage of the algorithm, while integration at the NSEv stage affected both interaction identification and the ability to simulate continuous gene expression levels. This suggests that staged evaluation integration (NSEv) is mandatory if improved quantitative performance is desired. Importantly, NSEv was considerably more sensitive to input data type, reflecting the more stringent integration criterion mechanism. While exploration (NSEx) seemed to benefit from the consideration of all data, even those with less clear information on direct gene interactions, NSEv performed best when only binding site affinity data were integrated with the basic series. This suggests that even quite noisy data can be used to drive the search the scope of different GRN models, but that evaluation of the model set obtained requires better precision, *i.e*., more reliable data. The analysis presented here provided us with an optimal integration strategy, which, when applied to both time series datasets (SC and DC), led to further improvement in gene interaction information.

The general methodology for the data integration presented is applicable to any process driven by gene expression. However, it has been tested only on *Drosophila melanogaster* embryo development and associated datasets available for this system. When changing the system under analysis, e.g., to explore a different process or organism, both data types available and their quality may also change, so a similar staged analysis is crucial to determining the optimal integration strategy.

## Supplementary Files

Supplementary File 1

## Abbreviations/Nomenclature

ANN: artificial neural network
AUROC: area under the ROC curve
AUPR: area under the precision-recall 120 curve
BSA: binding site affinity
CORR: correlations
DC: dual channel
DROID: Drosophila Interactions Database
EGIA: evolutionary computation for GRNs, an integrative algorithm
GEO: Gene Expression Omnibus
GO: Gene Ontology
GRN: gene regulatory network
KO: knock-out
NSEv: network structure evaluation
NSEx: network structure exploration
PSWM: position-specific weight matrix
RNA-seq: RNA sequencing
SC: single channel
